# A dataset of aligned RGB and multispectral UAV imagery for semantic segmentation of weedy rice

**DOI:** 10.1016/j.dib.2025.112237

**Published:** 2025-11-10

**Authors:** Van-Hoa Nguyen, Cong-Doan Le, Minh-Tuyen Truong, Mai-Phung Thi Bui, Thanh-Phong Le

**Affiliations:** a Faculty of Information Technology, An Giang University, 88000 An Giang, Vietnam; b Faculty of Engineering - Technology - Environment, An Giang University, 88000 An Giang, Vietnam; c Climate Change Institute, An Giang University, 88000 An Giang, Vietnam; d Vietnam National University Ho Chi Minh City, 70000-HCM, Vietnam

**Keywords:** Remote sensing, Precision agriculture, Image alignment, Spectral bands

## Abstract

This article introduces a curated UAV dataset for detecting and segmenting weedy rice in cultivated fields. It includes 734 high-resolution RGB images along with their geospatially aligned multispectral (MS) counterparts, which feature four spectral bands: Green, Red, Red Edge, and Near-Infrared. The RGB images were annotated with polygon masks created by a fine-tuned Segment Anything model, then reviewed and corrected by experts. All images were resized to 1280 × 960 pixels and further processed to remove samples with missing or excessively extensive annotations (>90 %). The final dataset reflects various infestation levels, with weedy rice coverage ranging from under 5 % to nearly 90 %, supporting the development of robust models in diverse field conditions. All images were acquired in rice fields across Vietnam's Mekong Delta region during three consecutive cropping seasons using consumer-grade UAVs equipped with both RGB and MS sensors. This dataset provides extensive spectral and spatial information, making it a valuable resource for research in precision agriculture, including multimodal semantic segmentation, vegetation classification, and weed detection. It also facilitates benchmarking and domain adaptation studies to improve model generalization across different modalities.

Specifications Table

**Table:** 

Subject	Computer Sciences
Specific subject area	Computer Vision, Precision Agriculture, and Geospatial Artificial Intelligence
Type of data	Aligned RGB images (JPEG, 2400 × 1900) Aligned multispectral images (TIFF, 2400 × 1900; Green, Red, Red Edge, NIR) Pixel-level ground-truth masks (PNG, 2400 × 1900) Metadata (CSV: spatial, temporal, and sensor parameters)
Data collection	UAV imagery was collected using a DJI Mavic 3 Multispectral (M3M) UAV equipped with a 4/3 RGB CMOS and 1/2.8-inch MS CMOS sensor. RGB images (5280 × 3956, JPEG) and spectral bands (2592 × 1944, TIFF: Green, Red, Eed Edge, NIR) were captured simultaneously at altitudes of 12 and 20 meters above ground level. The MS bands include Green (560 ± 16 nm), Red (650 ± 16 nm), Red Edge (730 ± 16 nm), and Near-Infrared (860 ± 26 nm). The images are georeferenced in WGS 84 / UTM zone 48 N and aligned at the pixel level. Metadata encompasses spatial, temporal, and sensor details for each image. Data were collected during three consecutive cropping seasons, from June 2024 to January 2025, in the Mekong Delta, Vietnam.
Data source location	City/Town/Province/Region: Thoaison District and Longxuyen City, An Giang Province, Mekong Delta Country: Vietnam GPS coordinates approx.: 10.38°N, 105.43°E
Data accessibility	Repository name: WeedyRice-RGBMS-DB [1] Data identification number: 10.17632/vt4s83pxx6.1 Direct URL to data: https:/data.mendeley.com/datasets/vt4s83pxx6/1 Instructions for accessing this data: freely available under a CC-BY 4.0 license; no login is needed.

## 1 Value of the Data

• This dataset offers a high-resolution, georeferenced RGB and MS imagery of rice fields infested with weedy rice in the Mekong Delta, Vietnam. It includes polygon-based annotations for semantic segmentation tasks, which are rarely available in public UAV agricultural datasets.
• The combination of RGB and four spectral bands (Green, Red, Red Edge, and Near-Infrared) provides rich spectral and spatial information to support the development of multimodal deep learning models in precision agriculture.
• The dataset directly benefits agricultural applications by enabling detection and spatial monitoring of weedy rice, thereby supporting site-specific weed management and reducing yield losses.
• The dataset, to our knowledge, represents the first publicly available UAV dataset on weedy rice with aligned RGB–MS imagery spanning multiple seasons, providing a comprehensive resource for research in computer vision and practical crop monitoring.

## 2 Background

• Weedy rice poses a major biological threat to sustainable rice cultivation in many Asian countries, including Vietnam [2]. Because of its close visual similarity to cultivated rice, early detection remains a technical challenge for both farmers and automated systems.
• Recent advancements in UAV platforms equipped with MS sensors now enable the collection of high-resolution spatial and spectral data from rice fields. These technologies create new opportunities for applying remote sensing and computer vision techniques to weed detection tasks.
• This dataset provides spatially aligned RGB–MS imagery with polygon-based ground-truth annotations of weedy rice in the Mekong Delta. It is intended to support research in geospatial AI and precision agriculture.
• In comparison, other UAV-based weed datasets, such as CoFly-WeedDB [3] on cotton in Greece and DRONEWEED [4] on tomato and maize in Spain, focus only on RGB imagery in different cropping systems (Table 1).

**Table 1:** Comparison of UAV-based weed datasets.

Dataset	Crop type	Sensor & modality	Annotation type	Geo. location
CoFly-WeedDB [3]	Cotton	UAV RGB	Bounding box	Greece
DRONEWEED [4]	Maize, Tomato	UAV RGB	Bounding box	Spain
WeedyRice-RGBMS-DB	Rice	UAV RGB and MS	Polygon segmentation	Vietnam

## 3 Data Description

The dataset is organized within the main directory WeedyRice-RGBMS-DB, which contains five subfolders and a README.md file that offers usage instructions and metadata. All images were captured by a DJI M3M UAV over rice fields in An Giang province, Vietnam, and are georeferenced in WGS 84/UTM zone 48 N. The folder structure and contents are as follows:

• RGB/: Contains RGB images in JPEG format (.JPG) with a resolution of 1280 × 960 pixels.
• Multispectral/: Includes aligned MS bands in TIFF format (.TIF), each with a resolution of 1280 × 960 pixels. Each image represents one of four spectral bands: Green (_G.TIF), Red (_R.TIF), Red Edge (_RE.TIF), and Near-Infrared (_NIR.TIF).
• Masks/: Contains binary ground-truth masks (.png) for semantic segmentation. Each mask is a single-channel (grayscale) image, where pixel values are 255 for weedy rice and 0 for background.
• Overlay/: Includes visualization overlays that superimpose masks onto the original images to support visual verification of segmentation quality.
• Metadata/: Includes a *filename_mapping.csv* file that maps original filenames to standardized names, and *an image_metadata.csv* file providing acquisition date and time, GPS coordinates, altitude, sensor type, and image band information for each image.

The folder structure and contents of the dataset are summarized in Table 2, which indicates the number of images and the file name format for each data type.

**Table 2:** Summary of dataset contents and file structure.

Folder	No. of images	Contents	Filename
RGB/	734	High-resolution RGB UAV images	DJI_XXXX.JPG
Multispectral/	2936	Pixel-aligned spectral bands (G, R, RE, NIR)	DJI_XXXX.TIF
Masks/	734	Ground-truth segmentation masks	DJI_XXXX.png
Overlay/	734	Visualization of masks over original images	DJI_XXXX.JPG
Metadata/	2	Image metadata and filename mappings	XXXX.csv

## 4 Experimental Design, Materials, and Methods

### 4.1 UAV flights and data acquisition

**Fig. 1:**
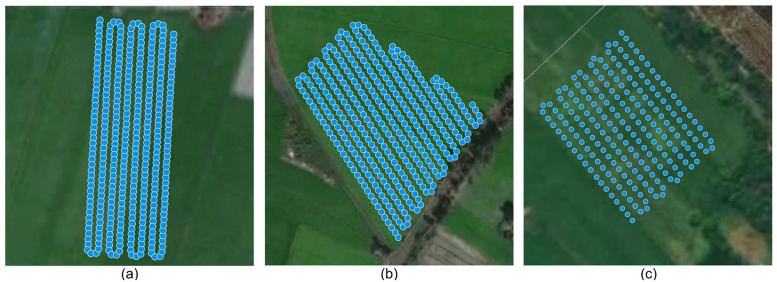
Spatial distribution of captured images during UAV missions over rice fields in Thoaison District: (a) coverage on June 2, 2024; (b) partial coverage on June 4, 2024; and (c) partial coverage on January 15, 2025, in Longxuyen City.

UAV-based image collection was conducted on four occasions: June 2, June 4, and September 30, 2024 (Thoaison District), and January 15, 2025 (Longxuyen City), all in An Giang Province, Vietnam. The schedules were set up to coincide with 55–60 days after sowing, when visual differences between cultivated rice and weedy rice become prominent. The spatial distribution of UAV-captured images over the rice fields is shown in Fig. 1.(blue dots), including missions on June 2 and partial coverage on June 4, 2024 (each covering approximately 2 hectares), and on January 15, 2025 (covering approximately 1 hectare).

The flights were performed between 09:30 a.m. and 3:30 pm. under clear daylight conditions. A DJI M3M UAV was utilized, flying at altitudes of 12 and 20 m above ground level, with 70 % front and side overlap to ensure high image quality and spatial coverage. Each RGB image was captured alongside four spectral bands (Green, Red, Red Edge, and Near-Infrared).

A total of 1471 RGB images and 5884 MS band images were obtained. 734 RGB images were selected for manual annotation based on visual clarity, crop stage visibility, and diversity of the field conditions. A summary of acquisition dates, locations, and selected images is included in Table 3.

**Table 3:** Summary of UAV data acquisition events, showing the number of collected RGB images and corresponding MS images, as well as the number of selected RGB images used for annotation at each location and date.

Date	Location	No. of RGB images	No. of MS images	Selected RGB images
02/06/2024	Thoaison	359	1436	115
04/06/2024	Thoaison	619	2476	331
30/09/2024	Thoaison	78	312	41
15/01/2025	Longxuyen	415	1660	247
Total	-	1471	5884	734

### 4.2 Data annotation

**Fig. 2:**
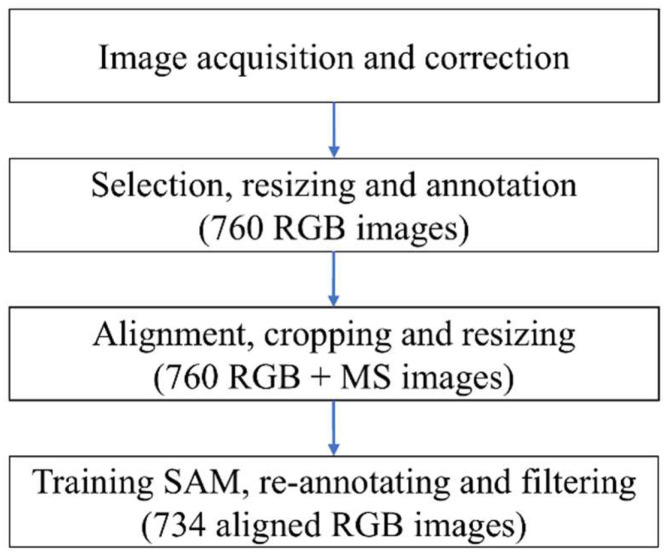
Diagram of data annotation.

The annotation process involved several sequential steps, from image correction to final mask refinement and quality filtering. An overview of this diagram is shown in Fig. 2, including RGB selection and annotation, MS alignment, and re-annotation.

#### 4.2.1 Correction and annotation

All RGB images captured by the DJI M3M UAV needed correction for optical and radiometric distortions caused by lens and lighting effects [5]. These correction steps addressed lens distortion, vignetting, geometric deformation, and exposure inconsistencies. Image undistortion was performed using intrinsic camera parameters, polynomial correction models, and onboard sensor data recorded in each image’s metadata.

Following preprocessing, 760 RGB images were selected from the initial set of 1471 images based on criteria such as high visual clarity, sufficient field coverage, and diversity in agronomic conditions, particularly with varying levels of weedy rice infestation. To ensure that segmentation masks captured meaningful contents, only images where annotated areas occupied >0 % and <95 % of the total image area were retained. All selected images were resized from their original resolution (5280 × 3956 pixels) to 1280 × 960 pixels to facilitate efficient manual annotation. Annotation was performed using the open-source tool LabelMe [6] by experts, with polygons drawn around visible patches of weedy rice. This set will then be used as the initial data for training the model.

#### 4.2.2 Multispectral alignment, cropping, and re-annotation

For each of the 760 RGB images selected, the original high-resolution RGB image was spatially aligned with its corresponding four MS bands, originally captured at 2592 × 1944 pixels, using feature-based image registration. First, the SIFT key-point detector [7] was implemented to extract features from a pair of a cropped RGB image and a reference spectral band, such as the Red band. Then, descriptor matching was applied to extracted key-points to filter the best matching ones, followed by the estimation of an affine transformation to align the RGB image with the reference band [8].

The remaining spectral bands (Green, Red Edge, and NIR) were then geometrically aligned to the same reference, resulting in five spatially registered images (aligned RGB and four spectral bands) per instance. To ensure consistent dimensions and eliminate edge artifacts, all aligned images were cropped to remove black borders, resulting in a size of 2400 × 1800 pixels, which allows for precise pixel-level correspondence across modalities.

**Fig. 3:**
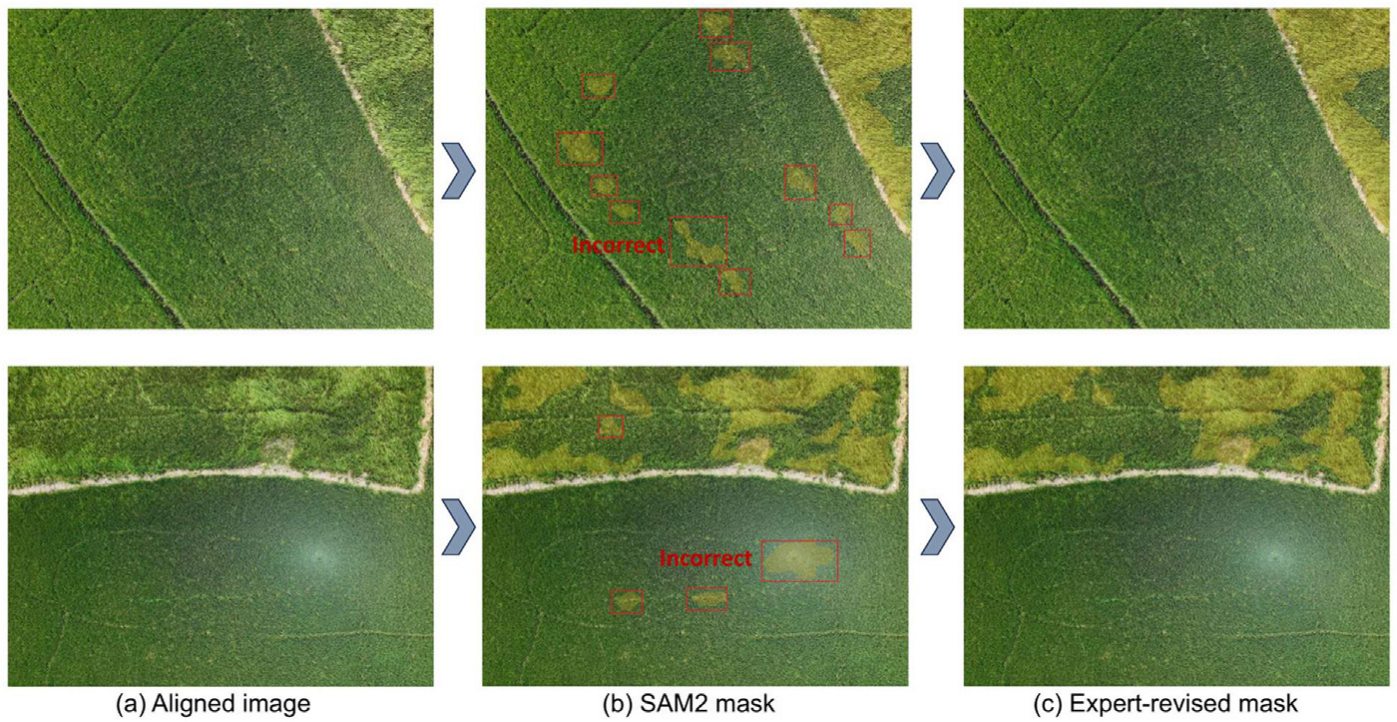
Example of annotation workflow: (a) aligned RGB image, (b) initial SAM2-generated mask (with incorrect regions highlighted), and (c) expert-revised mask.

Subsequently, all aligned RGB and MS images were resized to a standard resolution of 1280 × 960 pixels to ensure consistency in the input for model training. The Segment Anything model (SAM) [9] was fine-tuned using the initial set of 760 manually annotated RGB images. This trained model was then used to generate automatic annotations for the spatially aligned RGB images, followed by manual review and correction by experts to ensure the quality of the labels. An example of this annotation workflow (original image → SAM2 mask → expert-revised mask) is illustrated in Fig. 3.

**Fig. 4:**
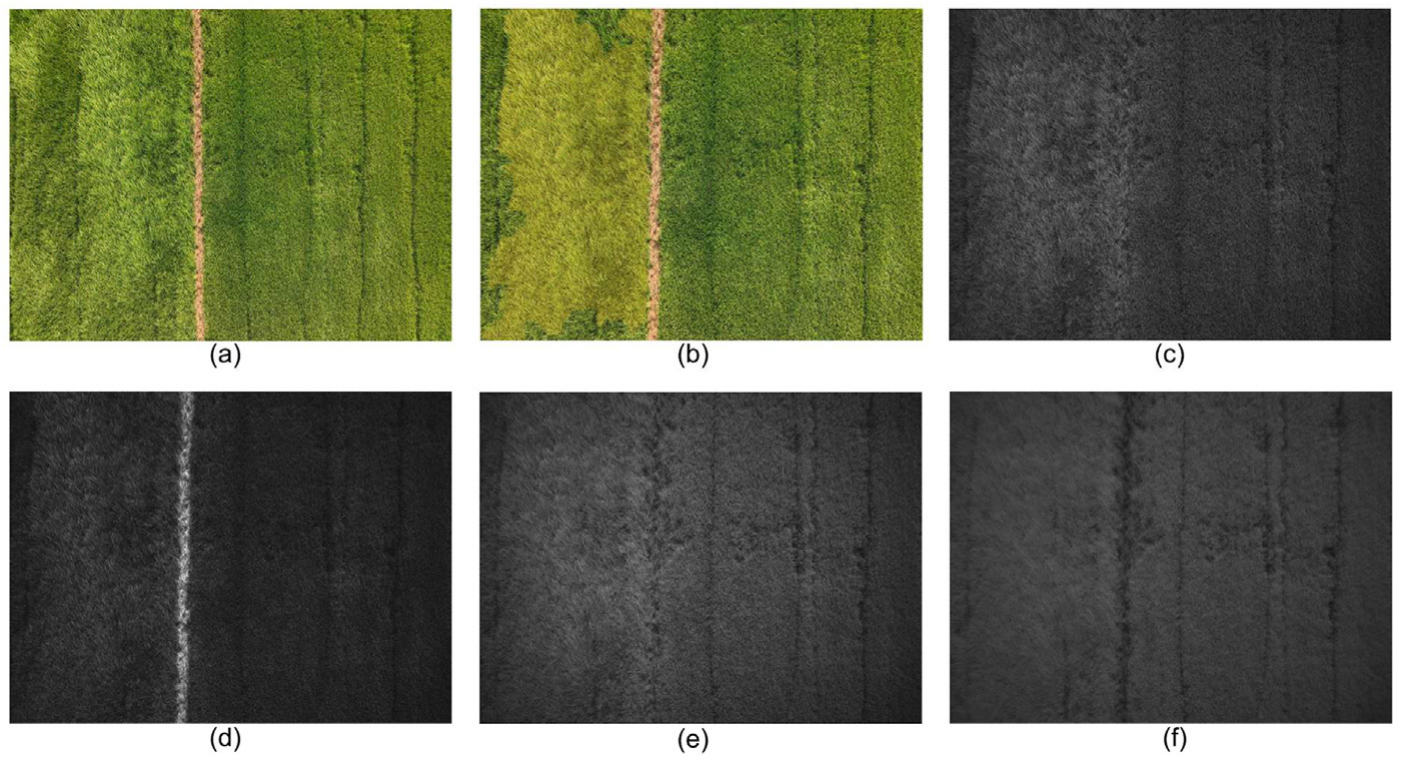
Illustration of the RGB-to-MS alignment and mask overlay process. (a) Original high-resolution RGB UAV image before cropping; (b) RGB image after cropping and alignment, with an overlaid segmentation mask for weedy rice; (c–f) Corresponding aligned spectral bands: Green, Red, Red Edge, and Near-Infrared.

A final quality filtering step was performed after re-annotation. Out of 760 annotated images, 734 were retained based on a stricter criterion: annotated regions must cover >0 % and <90 % of the entire image. The remaining 26 images were discarded due to excessive label coverage. The final dataset includes 734 RGB images and their aligned MS images, all resized to a consistent resolution of 1280 × 960. Fig. 4 provides a visual example of the image preprocessing steps, showing the original RGB image, the cropped and aligned RGB image with mask overlay, and the corresponding aligned MS images.

Table 4 presents the distribution of these images according to the percentage of weedy rice pixels. Most images fall within the 10–20 % and 40–60 % coverage ranges, with an average coverage of 31.7 % across all images, supporting robust model development across different conditions.

**Table 4:** Distribution of weedy rice coverage across images.

Range of Weedy Rice Pixels (%)	Number of Images	Percentage (%)
>0–5	71	9.7
>5–10	88	12.0
>10–20	171	23.3
>20–30	79	10.8
>30–40	66	9.0
>40–60	151	20.6
>60–75	43	5.9
>75–90	65	8.9
*Average*	*-*	*31.7*

## Limitations

While the dataset offers high-resolution RGB and aligned MS images with detailed annotations of weedy rice infestations, several limitations should be acknowledged.

• First, image collection was limited to a specific region and cropping seasons in the Mekong Delta, which may restrict the dataset's usefulness in other geographic areas.
• Second, the quality of annotations depends on how visual symptoms appear in UAV images; in some cases, slight differences between weedy rice and cultivated rice can lead to uncertainty in annotations.
• Third, filtering based on mask coverage thresholds (0–90 %) might exclude extreme infestation cases, which could be helpful in different contexts.
• Lastly, although the dataset includes 734 image pairs, class imbalance remains an issue since images with moderate infestation are more frequent than those with very low or very high coverage.

## Ethics Statement

This work does not involve human subjects, animal experiments, or data from social media platforms.

## Credit Author Statement

**Van-Hoa Nguyen:** Conceptualization, Methodology, Writing – Review, Project administration. **Cong-Doan Le:** Software, Data curation, Writing – Review & Editing. **Minh-Tuyen Truong:** Software, Data curation. **Mai-Phung Thi Bui:** Data curation, Validation. **Thanh-Phong Le:** Investigation, Data curation, Validation.
